# ESRP1 Induces Cervical Cancer Cell G1-Phase Arrest Via Regulating Cyclin A2 mRNA Stability

**DOI:** 10.3390/ijms20153705

**Published:** 2019-07-29

**Authors:** Zhi-Hong Chen, Ya-Jie Jing, Jian-Bo Yu, Zai-Shu Jin, Zhu Li, Ting-Ting He, Xiu-Zhen Su

**Affiliations:** 1 School of Basic Medicine, Youjiang Medical University for Nationalities, No. 98 Chengxiang Road, Baise 533000, China; 2 Heilongjiang Province Key Laboratory of Cancer Prevention and Treatment, Mudanjiang Medical University, No. 3, Tongxiang Street, Mudanjiang 157011, China; 3 Sciences Research Center, Youjiang Medical University for Nationalities, No. 98 Chengxiang Road, Baise 533000, China

**Keywords:** ESRP1, G1-phase arrest, cyclin A2, mRNA stability, cell proliferation

## Abstract

Accumulating evidence indicates that epithelial splicing regulatory protein 1 (ESRP1) can inhibit the epithelial-to-mesenchymal transition (EMT), thus playing a central role in regulating the metastatic progression of tumors. However, it is still not clear whether ESRP1 directly influences the cell cycle, or what the possible underlying molecular mechanisms are. In this study, we showed that ESRP1 protein levels were significantly correlated with the Ki-67 proliferative index (*r* = −0.521; *p* < 0.01), and that ESRP1 overexpression can significantly inhibit cervical carcinoma cell proliferation and induced G1-phase arrest by downregulating cyclin A2 expression. Importantly, ESRP1 can bind to GGUGGU sequence in the 3′UTR of the cyclin A2 mRNA, and ESRP1 overexpression significantly decreases the stability of the cyclin A2 mRNA. In addition, our experimental results confirm that ESRP1 overexpression results in enhanced CDC20 expression, which is known to be responsible for cyclin A2 degradation. This study provides the first evidence that ESRP1 overexpression induces G1-phase cell cycle arrest via reducing the stability of the cyclin A2 mRNA, and inhibits cervical carcinoma cell proliferation. The findings suggest that the ESRP1/cyclin A2 regulatory axis may be essential as a regulator of cell proliferation, and may thus represent an attractive target for cervical cancer prevention and treatment.

## 1. Introduction

Cervical cancer (CC) is among the most frequently encountered forms of gynecological malignancies, resulting from the abnormal malignant proliferation of cervical epithelial cells. At present, due to the complexity of the regulatory mechanisms governing tumor cell proliferation, the etiology and pathogenesis of this disease remain unclear. Thus, investigating the mechanisms regulating the cell cycle will help to clarify the molecular mechanisms underlying the genesis and development of CC and provide more clues for the clinical prevention and treatment of CC, as well as helping to guide novel drug design [1,2].

The cell cycle governs cellular proliferation, progressing through the G1, S, G2, and M -phases in a carefully regulated and ordered fashion [3]. Cell cycle checkpoints exist to help regulate the order of cell cycle transitions, and to guarantee that DNA is properly replicated and cells are prepared to divide, with G1, G2, and metaphase (M) or spindle checkpoints [4]. In the mammalian cell cycle, the G1 checkpoint is referred to as a restriction point (R-point), as it represents the checkpoint after which cells commit to enter into the S-phase, ultimately leading to cell cycle completion [5,6]. When cells fail to pass through the R-point, the G1-to-S-phase transition fails to occur, leading to G1-phase arrest, and many studies have confirmed that cyclin A2, a key cell cycle regulator, initiates and controls DNA replication, which is crucial for DNA synthesis and for the G1/S-phase transition. Among the most common types of cancer, cyclin A2 is considered to be a major factor controlling cell proliferation, and increased expression of cyclin A2 protein has been observed in many types of cancer such as breast, cervical, liver, and lung cancer, with only limited expression in normal tissues [4,7,8].

RNA recognition motif (RRM)-type RNA-binding proteins (RBPs) are RNA binding proteins with one or more RRM domains and accessory domains, which participate in many post-transcriptional processes, including the splicing of RNA precursors, as well as the localization and stability of RNAs within the cell [9,10]. Recent work indicates that RRM-type RBPs play a key role in the cell cycle process. For example, Human antigen R (HuR) can participate in cell cycle regulation via binding to the 3′UTR (untranslated region) of cyclin D or p27 mRNAs, regulating their expression by stabilizing the mRNA at the post-transcriptional level [11,12].

Epithelial splicing regulatory protein 1 (Esrp1) is an RRM-type RBP, expressed in epithelial cells, that is composed of three RRM domains, and which functions via regulating epithelial cell-specific mRNA isoform splicing [13]. It regulates alternative splicing during the epithelial-mesenchymal transition (EMT), a process which leads to tumors becoming more invasive and, thus, acts as a key step in tumor metastasis in many cancer types—such lung cancer, breast cancer, and colon cancer. Tumor inhibitors acting at this stage can prevent or slow cancer invasion and metastasis [14,15,16]. ESRP1 mediates the alternative splicing of CD44, CTNND1, ENAH, and FGFR2, among other genes, by directly binding specific GU-rich sequence motifs in the mRNAs encoding these proteins, thereby allowing it to modulate the EMT. In addition to EMT, it has been reported that ESRP1 can inhibit tumor cell proliferation, but the exact cellular and molecular mechanisms underlying ESRP1-mediated inhibition of cell proliferation remain unknown [17].

Herein, we aimed to determine the importance of ESRP1 in cervical carcinoma and to explore the molecular mechanisms whereby it inhibits cell proliferation, clarifying the role of ESRP1 as a potential tumor suppressor gene in cervical cancer. Our results showed that ESRP1 is essential for cyclin A2 mRNA stability, and for the G1/S transition. Our results not only highlight the molecular mechanisms of ESRP1 in the context of tumor cell proliferation, but further provide new insights with respect cyclin A2 post-transcriptional regulation.

## 2. Results

### 2.1. Immunohistochemical Assessment of ESRP1 Levels in Human Cervical Carcinoma Tissues

**Figure 1:**
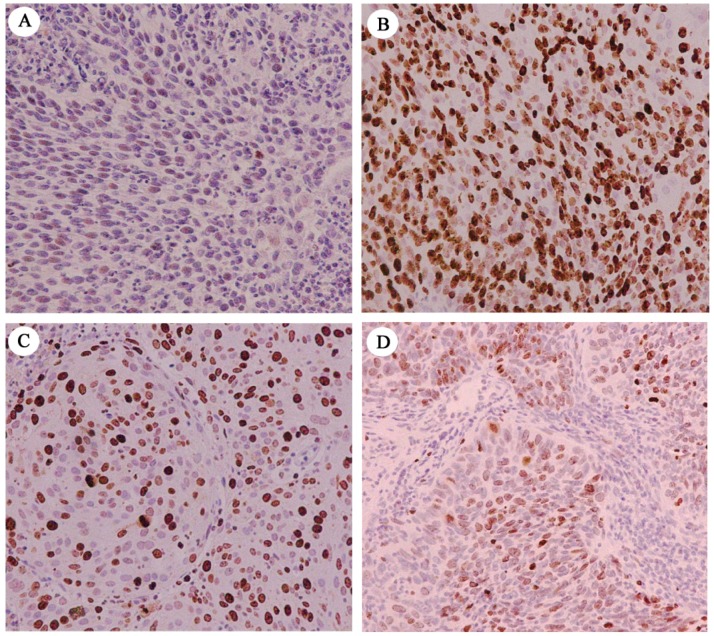
Expression of ESRP1 and Ki-67 in human cervical carcinoma tissues. (**A**,**B**) Low expression of ESRP1 and high expression of Ki-67 in a single cervical carcinoma sample, respectively. (**C**,**D**) High expression of ESRP1 and low expression of Ki-67 in another cervical carcinoma sample, respectively. (200×).

We first began by assessing ESRP1 protein levels, as well as those of the widely used proliferative index Ki-67 [18], in 44 archived paraffin-embedded human cervical carcinoma tissue samples via immunohistochemical staining (Figure 1). We observed high and low ESRP1 protein levels of 38.6% (17/44) and 61.4% (27/44), respectively. High and low expression rates for Ki-67 were 52.3% (23/44) and 47.7% (21/44), respectively, in these same samples. In addition, we found that protein levels of ESRP1 were significantly negatively correlated with those of Ki-67 (*r* = −0.521; *p* < 0.01). This result suggests that the splicing factor ESRP1 may be a key regulatory factor in the context of human cervical carcinoma cancer progression, negatively regulating tumor cell proliferation.

### 2.2. ESRP1 Overexpression Inhibits Cervical Carcinoma Cell Proliferation Via Inducing G1-Phase Arrest

**Figure 2:**
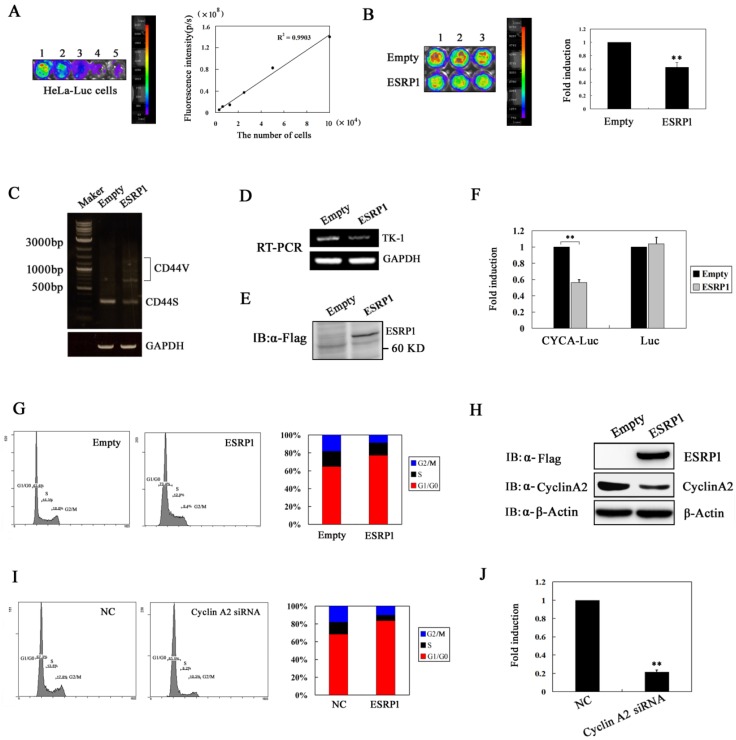
ESRP1 overexpression inhibits the proliferation of HeLa cells via inducing G1-phase arrest. (**A**) HeLa-Luc cells were serially diluted, placed into the wells of a 96-well plate, and immediately imaged using the NightOWL LB 983 system to obtain FLUX measurements per well. (**B**) HeLa-Luc cells were seeded into 96-well plates, and 48 h after transfection with pcDEF-FLAG-ESRP1(ESRP1) or a control vector (empty vector) (0.2 μg/well), FLUX measurements were captured (left). For normalization of luciferase activity, the luciferase signal per well of the control cells was set to 1. Quantitative data represent the mean ± standard error (*n* = 3 per group) (right). HeLa cells were seeded into 6-well plates, and 48 h after transfection with ESRP1 or a control vector (2 μg/well), CD44 isoforms (**C**) or TK-1 expression (**D**) was measured via RT-PCR in HeLa cells following overexpression of ESRP1, with GAPDH as a control. ESRP1 levels in HeLa cells were measured via Western blotting, with empty vectors serving as negative controls (**E**). (**F**) 48 h after ESRP1 vector or empty transfection, HeLa-CYCA-Luc or HeLa-Luc cells were lysed, and luciferase activity was assessed. (**G**) The cell cycle distribution in ESRP1-overexpressing HeLa cells and empty control groups was assessed via flow cytometry, revealing G1-phase arrest in HeLa cells 48 h after transfection with ESRP1 vector. (**H**) ESRP1 or cyclin A2 levels in HeLa cells as measured by Western blotting. 48 h after treatment with 50 nM cyclin A2 siRNA for 48 h, HeLa-CYCA-Luc cells were analyzed via flow cytometry (**I**), or lysed and luciferase activity was assessed (**J**). For normalization of CYCA-Luc activity or Luc, the signal (1 μg protein) for control cells was set to 1. This experiment was repeated three times (*n* = 3); error bars indicate standard error; ** *p* < 0.01.

The impact of ESRP1 overexpression on the proliferation of cervical cancer cells was examined using a bioluminescence imaging approach. HeLa cells constitutively expressing luciferase (HeLa-Luc) were transiently transfected and cell number monitored through changes in signal intensity. ESRP1 overexpression caused decreased luciferase signals in transfected HeLa-Luc cell populations compared with cells transfected with empty expression vector, with an approximately one-fold decrease in signal 48 h post-transfection (Figure 2A,B). This indicates that ESRP1 overexpression can inhibit the growth of HeLa cervical carcinoma cells in vitro. To confirm this result, we further analyzed the expression of thymidine kinase 1 (TK-1) or CD44 isoforms in our system, as TK-1 is known to be a tumor proliferation marker [19], and ESRP1 is known to regulate CD44 isoform switching during the EMT [20]. We found that cells overexpressing ESRP-1 exhibited significantly reduced TK-1 expression as compared to the control vector, and ESRP1 overexpression inhibited isoform switching from CD44v to CD44s (Figure 2C–E). These data suggest that ESRP1 acts to inhibit tumor cell proliferation and the EMT in human cervical carcinoma cancer cells.

To assess how ESRP1 overexpression affects cell cycle progression, we conducted luciferase activity assays and flow cytometric analyses. Specifically, we employed the CYCA-Luc fusion reporter system as a means of monitoring cell entry into the cell cycle S-phase, and to assess endogenous cyclin A2 levels, as we have reported previously [21]. HeLa-CYCA-Luc cells, stably expressing the fusion protein cyclinA2-luciferase (CYCA-Luc), or HeLa-Luc cells, were transiently transfected using an ESRP1 or empty control vector, after which luciferase activity was assessed. We found that ESRP1 overexpression significantly decreased CYCA-Luc luciferase activity, whereas this activity was unaffected in HeLa-Luc cells, suggesting that the cell cycle distribution could be changed by ESRP1 overexpression (Figure 2F). Moreover, FACS analysis revealed that ESRP1 overexpression resulted in an increase of HeLa cells in the G1-phase from 63.6% to 73.4%, relative to controls (Figure 2G). We then further assessed endogenous cyclin A2 protein levels. As expected, ESRP1 overexpression resulted in downregulation of the key G1/S transition protein cyclin A2, (Figure 2H). Repression of cyclin A2 expression has been reported to result in cycle G1-phase arrest [22], and cyclin A2 siRNA treatment is known to result in cell cycle G1-phase arrest in HeLa cervical cancer cells, as was confirmed in our experiments (Figure 2I,J). Taken together, these data suggested that the ESRP1 may induce G1-phase arrest in human cervical cancer cells by downregulating cyclin A2 expression.

### 2.3. ESRP1 Regulates the Stability of the Cyclin A2 mRNA Via 3′UTR Interactions

**Figure 3:**
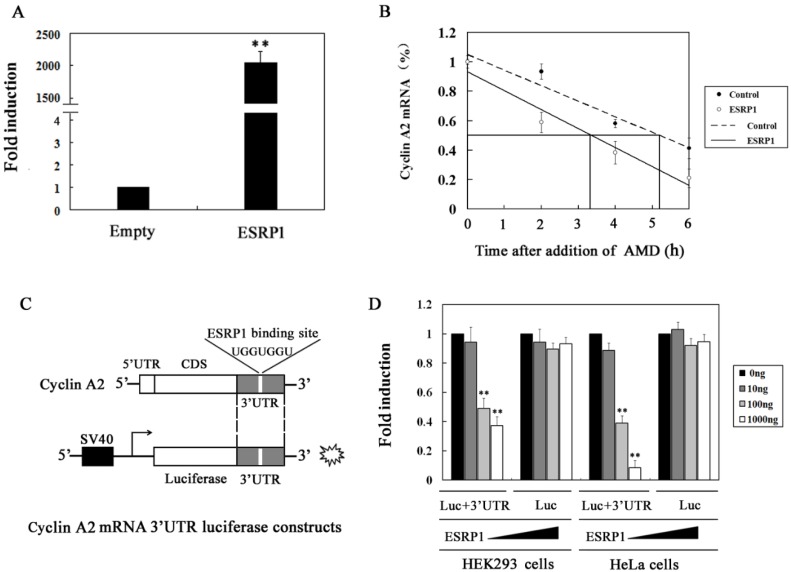
ESRP1 overexpression leads to decreased cyclin A2 mRNA stability. (**A**) 48 h after transfection with an ESRP1 vector, ESRP1 expression in HeLa cells was detected by qRT-PCR. (**B**) 24 h following ESRP1 vector transfection, cells were treated using 5 g/mL actinomycin D (AMD), with RNA being isolated at the indicated times, after which cyclin A2 expression was determined via qRT-PCR. (**C**) Schematic diagram of the construction of a luciferase-cyclin A2 3′UTR chimeric vector generated via subcloning the cyclin A2 3′UTR region into the pEZX-MT06 vector encoding firefly luciferase. (**D**) HeLa cells or HEK293 cells in 24-well plates were co-transfected with full-length vector or control vector, along with ESRP1 vector (0, 10, 100, and 1000 ng), and after 48 h, cells were lysed and luciferase activity was assessed. For normalization of luciferase activity, the signal (1 μg protein) for control cells was set to 1. This experiment was repeated three times (*n* = 3); error bars indicate standard error; ** *p* < 0.01.

We next assessed the mechanisms whereby ESRP1 overexpression led to reduced cyclin A2 expression. Previous studies have documented that certain RNA-binding proteins are able to bind to mRNAs encoding cyclins A and B1, thereby bolstering their stability [23]. As ESRP1 is an RNA-binding protein, we sought to determine whether it post-transcriptionally regulates cyclin A2 expression via interacting with the cyclin A2 mRNA 3′UTR region. To answer this question, we first assessed the cyclin A2 mRNA half-life. To this end, 24 h following ESRP1 transfection, we treated cells using 5 μg/mL actinomycin D and then isolated RNA at appropriate time points to assess whether ESRP1 influenced the rate of cyclin A2 mRNA degradation. In cells overexpressing ESRP1, we observed a marked decreased in cyclin A2 mRNA half-life from about 5 to 3 h, relative to control cells (Figure 3A,B). We additionally constructed a wild-type 3′UTR reporter gene vector (Figure 3C), and analyzed cyclin A2 transcription in cultured HeLa cells or HEK293 cells via a luciferase activity assay. As expected, ESRP1 led to a dose-dependent inhibition of luciferase activity produced from reporter constructs containing the cyclin A2 mRNA 3′UTR, whereas luciferase activity produced from control vector, pEZX-MT06, did not change significantly (Figure 3D). These results demonstrate that ESRP1 regulates cyclin A2 mRNA stability via interacting with the cyclin A2 mRNA 3′UTR.

### 2.4. ESRP1 Binds to the 3′UTR of Cyclin A2 mRNA

**Figure 4:**
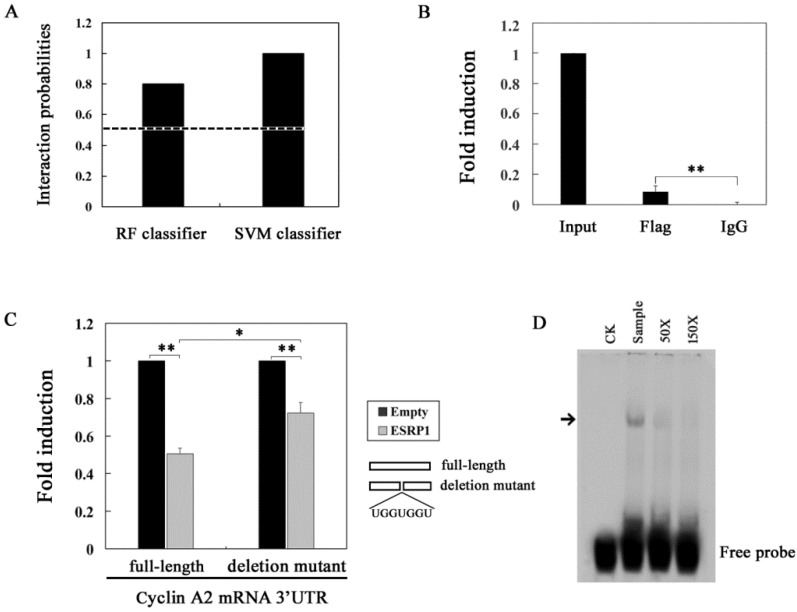
ESRP1 binds the cyclin A2 mRNA 3’UTR. (**A**) RPISeq was used to predict the likelihood of interactions between ESRP1 and the cyclin A2 mRNA, with any probabilities greater than 0.5 being deemed “positive”, suggesting a likely interaction. (**B**) RIP was performed using antibodies against FLAG in ESRP1-overexpressing HeLa cells, with ESRP1-bound transcripts being analyzed via qRT-PCR. (**C**) HEk293 cells in 24-well plates were co-transfected with full-length or deletion mutant vector (0.5 μg/well), along with ESRP1 vector (0.1 μg/well), and after 48 h cells were lysed and luciferase activity was assessed. (**D**) EMSA was performed using RNA transcript probes and FLAG -ESRP1. Arrowheads indicate the RNA-protein complexes. Competition experiments with a 50- and 150-fold excess of unlabeled RNA transcript probes were also performed. For normalization of luciferase activity, the signal (1 μg protein) for control cells was set to 1. This experiment was repeated three times (*n* = 3); error bars indicate standard error; * *p* < 0.05, ** *p* < 0.01.

To further explore the interactions between ESRP1 and the cyclin A2 mRNA, we utilized a bioinformatics tool (http://pridb.gdcb.iastate.edu/RPISeq/) to predict the interactions between these two molecules. This approach yielded Random Forest (RF) classifier and support vector machine (SVM) classifier scores of 0.8 and 1, respectively, suggestive of a likely binding interaction of cyclin A2 mRNA and ESRP1 (Figure 4A). Next, we performed an RNA immunoprecipitation (RIP) assay with a FLAG antibody, revealing that ESRP1 can bind to the cyclin A2 mRNA 3′UTR (Figure 4B). Previous research has established that ESRP1 recognition sequences contain UGG or GGU repeats [24], and we found that there was an ESRP1 recognition sequence: UGGUGGU in the 3′UTR of the cyclin A2 mRNA. To confirm whether this region was targeted by ESRP1 to mediate mRNA stability, we constructed a deletion mutant (deleting the UGGUGGU sequences) reporter gene vector, and utilized it for a luciferase activity assay. As shown in Figure 4C, this deletion mutant partly reduced the observed reduction in luciferase activity upon ESRP1 overexpression. This suggests that the UGGUGGU sequence is important for the decreased cyclin A2 mRNA stability caused by overexpression of ESRP1. To further assess how ESRP1 interacts with the cyclin A2 3′UTR, we conducted an EMSA experiment utilizing purified FLAG-ESRP1, together with in vitro synthesized transcripts. FLAG -ESRP1 protein was combined with biotin-labeled cyclin A2 3′UTR transcripts, and we then assessed these samples to detect RNA-protein complexes. We found that FLAG-ESRP1 bound the cyclin A2 3′UTR, while no control binding was observed (Figure 4D). Taken together, these results demonstrate that ESRP1 regulates cyclin A2 mRNA stability via interacting with the 3′UTR of this mRNA.

### 2.5. ESRP1 Downregulates Cyclin A2 Expression Partially Dependent on CDC20

**Figure 5:**
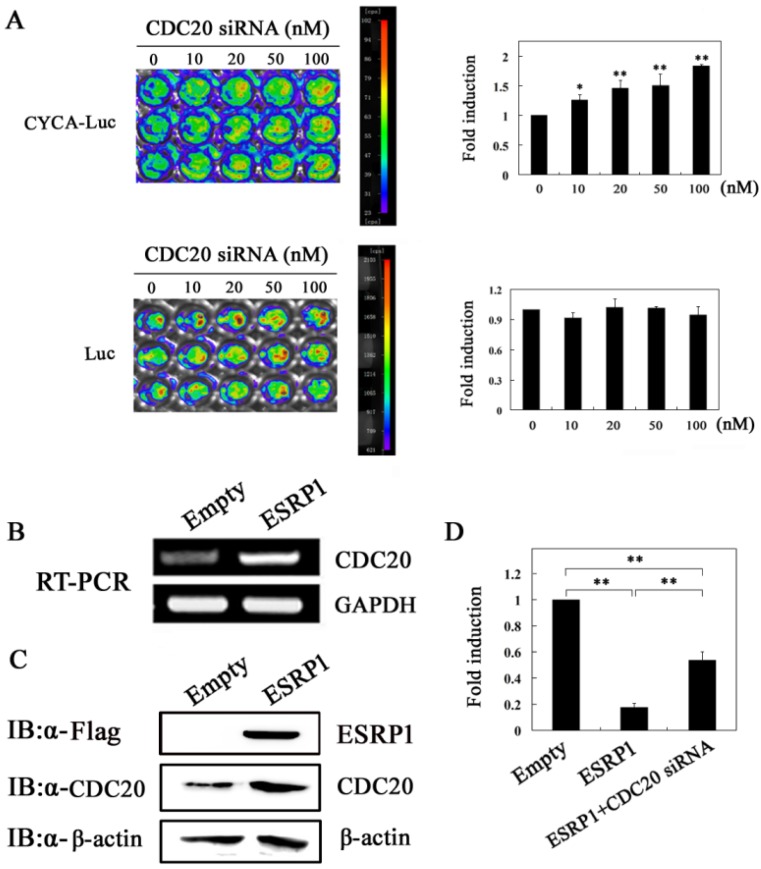
ESRP1 can increase the expression of CDC20 by inducing G1-phase arrest. (**A**) HeLa-Luc cells or HeLa-CYCA-Luc cells were seeded in 96-well plates, treated with CDC20 siRNA (0, 10, 20, 50, and 100 nM), and then imaged after 48 h. Images of siRNA-treated cells are shown on the left, whereas the normalized fold induction in luciferase signal activity upon siRNA treatment is shown on the right, with 1 corresponding to activity in untreated cells. Quantitative data represent the mean ± standard error (*n* = 3 per group). Expression of CDC20 was assessed via RT-PCR (**B**) or Western blotting (**C**) in HeLa cells after overexpression of ESRP1, with an empty plasmid used as a negative control. (**D**) Hela-CYCA-Luc cells were transfected with the ESRP1 vector alone or together with CDC20 siRNA for 48 h, with empty vectors serving as negative controls, and then cells were lysed, and luciferase activity was measured. For normalization of CYCA-Luc activity, the signal (1 μg protein) for control cells was set to 1. This experiment was repeated three times (*n* = 3); error bars indicate standard error. * *p* < 0.05, ** *p* < 0.01.

CDC20 is an activator of the anaphase-promoting complex/cyclosome (APC/C), which forms the E3 ubiquitin ligase complex APC/C^CDC20^ by activating the APC, which is involved in the degradation process of downstream substrates, such as cyclin A2, thereby regulating the cell cycle progression [25,26,27]. In previous studies, we have demonstrated that APC/C^CDC20^ is responsible for the degradation of cyclin A2 in HeLa cells [21]. Therefore, the upregulated expression of CDC20 will lead to the decrease of cyclin A2 protein. So, can ESRP1 upregulate CDC20 expression? To clarify the question, in this experiment, we first observed the effect of siRNA-mediated CDC20 knockdown on the luciferase signal in HeLa-Luc or HeLa-CYCA-Luc cells. As expected, CDC20 siRNA treatment resulted in a dose-dependent increase in luciferase activity in HeLa-CYCA-Luc but not HeLa-Luc cells (Figure 5A), suggesting that CDC20 was responsible for cyclin A2 degradation, which is consistent with previous reports [21]. We then examined the effect of ESRP1 overexpression on CDC20 expression at the protein and mRNA level in HeLa cells, revealing that ESRP1 overexpression resulted in enhanced CDC20 expression (Figure 5B,C). We further examined the effect of downregulation of CDC20 on cyclin A2 expression induced by ESRP1 overexpression using HeLa-CYCA-Luc cells, revealing that CDC20 siRNA treatment partly eliminated the inhibitory effect of the over-expression of ESRP1 on CYCA-Luc luciferase activity, suggesting that CDC20 may be partly responsible for ESRP1-mediated downregulation of cyclin A2 expression (Figure 5D). These results showed that the downregulation of cyclin A2 expression by ESRP1 may be partly attributed to the up-regulation of CDC20 expression.

## 3. Discussion

This study provides novel evidence that ESRP1 can induce tumor cell G1-phase cell cycle arrest by regulating cyclin A2 mRNA stability. This crucial finding indicates that ESRP1 can not only inhibit the EMT by regulating alternative splicing, but can also inhibit the proliferation of tumor cells by regulating mRNA stability in tumor progression, suggesting that ESRP1 may be an important candidate target for future cancer treatment efforts.

Abnormal cell proliferation is a hallmark of tumors, and it provides key insights useful for the clinical evaluation of tumor malignancy [28]. High levels of ESRP1 protein have previously been observed in well-to-moderately differentiated cancer cells, whereas this expression was markedly reduced when cells were instead poorly-differentiated [29]. As poorly differentiated tumors have a higher proliferation rate than do well-differentiated tumors, we hypothesized that ESRP1 may be associated with malignant tumor cell proliferation. To confirm this hypothesis, in the present study, we first examined ESRP1 and Ki-67 expression in 44 human cervical cancer tissue samples, with Ki-67 used as a reliable proliferation marker. We observed a significant negative correlation between ESRP1 levels and the Ki-67 proliferation index in these tumor samples (*r* = −0.521; *p* < 0.01). This result strongly implies that ESRP1 is closely associated with malignant tumor cell proliferation, potentially playing a role in inhibiting human cervical carcinoma cell proliferation.

Cyclin A2 is well-known to be expressed primarily during the S-phase of the cell cycle, mediating the S-phase initiation and transit [30]. Inhibiting the expression of this cyclin protein can lead to G1-phase arrest in cells, as described above. In our previous work, we have developed a cyclin A2-luciferase (CYCA-Luc) fusion protein as a luminescent reporter that was confirmed to be regulated in a cell cycle dependent manner, with its regulation controlled by the APC/C^CDC20^-mediated ubiquitin-proteasome pathway in HeLa cells [21]. Because the regulation of CYCA-Luc mimics endogenous cyclin A2, CYCA-Luc activity can accurately reflect endogenous cyclin A2 expression, and therefore in this study we used this reporter to examine to dynamic changes in endogenous cyclin A2 or G/S phase transition. Using this model system, we found that ESRP1 overexpression significantly decreased CYCA-Luc luciferase activity, and we further found that that ESRP1 overexpression led to a downregulation of the expression of cyclin A2, and an induction of G1-phase arrest in HeLa cells. Furthermore, we also observed that ESRP1 overexpression led to an upregulation in the expression of CDC20, and that APC/C^CDC20^ was responsible for cyclin A2 degradation, which is consistent with previous observations [21]. When CDC20 expression was reduced via siRNA, ESRP1-mediated decreases in CYCA-Luc luciferase activity were partially rescued, suggesting that CDC20 may be partly responsible for ESRP1-mediated cell cycle G1-phase arrest. However, the molecular mechanism of upregulation of CDC20 by ESRP1 is unclear, which needs further study to elucidate.

**Figure 6:**
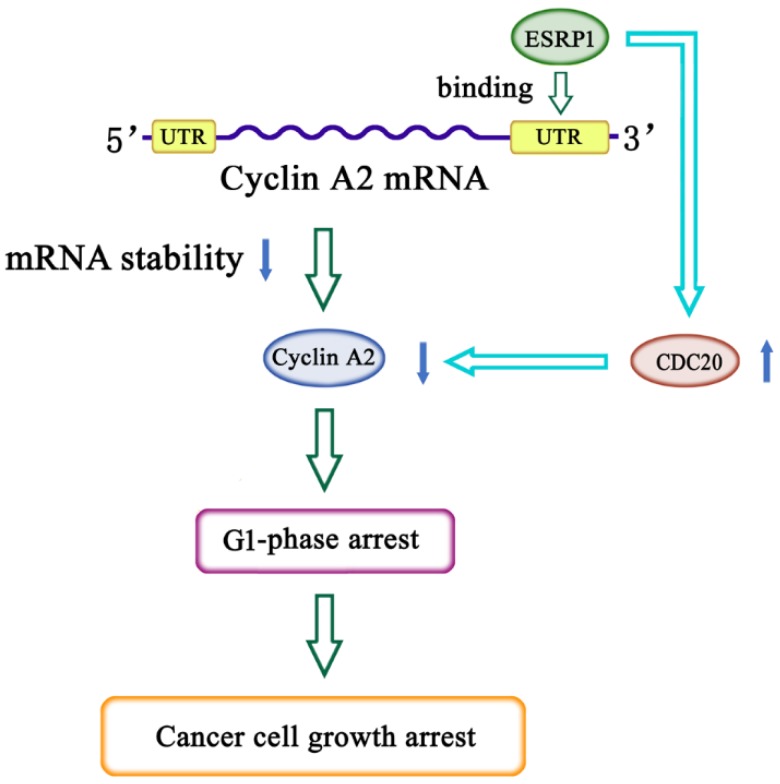
Model of ESRP1-mediated cell cycle G1-phase arrest. ESRP1 directly binds to the cyclin A2 mRNA 3′UTR, which decreases the stability of the cyclin A2 mRNA and downregulates cyclin A2 protein levels. Furthermore, ESRP1 can upregulate the expression of CDC20, which leads to further downregulation of cyclin A2 expression via the APC^CDC20^-mediated ubiquitin degradation pathway in HeLa cells.

RRM-type RBPs can participate in cell cycle regulation by modulating the stability of cycle-related mRNAs, as discussed above. It has been reported that ESRP1 selectively binds to UGG/GUU-rich sequence motifs and regulates the expression of EMT-related genes such as FGFR-2, Rac1b, and CD44 via alternative splicing [31,32,33]. In this study, we found that there are UGG/GUU-rich sequences in the cyclin A2 mRNA 3′UTR region, and RIP analyses also revealed that ESRP1 can interact with these regions. We therefore sought to assess whether ESRP1 may reduce cyclin A2 mRNA stability via binding its 3′UTR, leading to the downregulation of cyclin A2 protein expression at the post-transcriptional level and inducing cycle G1-phase arrest. RIP and EMSA analyses confirmed that ESRP1 was indeed able to directly bind to cyclin A2 mRNA 3′UTR, suggesting that this interaction underlies the observed changes in cyclin A2 mRNA stability. Even so, more work will be needed to determine whether other proteins also contribute to this control of cyclin A2 mRNA stability. Via the use of deletion constructs, we were able to determine that the UGGUGG was essential in order for ESRP1 to modulate cyclin A2 mRNA stability. This is the first report to date highlighting the importance of this sequence for the stability of the cyclin A2 mRNA. Nevertheless, our results showed that deletion of the UGGUGGU sequence did not completely abolish the inhibitory effect of the overexpression of ESRP1 on luciferase activity, suggesting that besides the UGGUGGU sequence, there are other binding sites in the cyclin A2 3′UTR region, which need to be identified in further research. Based on these results, we speculate that ESRP1 may induce G1-phase arrest by downregulating cyclin A2 expression via a dual mechanism. First, ESRP1 directly can bind to cyclin A2 mRNA 3′UTR and downregulate cyclin A2 expression by regulating the mRNA stability. Second, ESRP1 can upregulate the expression of CDC20, and cyclin A2 protein can be degraded by the APC/C^CDC20^ mediated ubiquitin-proteasome pathway (Figure 6).

In summary, we have demonstrated that ESRP1 overexpression induces G1-phase cell cycle arrest via reducing the stability of the cyclin A2 mRNA. This finding offers new insights into the molecular mechanisms of tumor cell cycle control. It is well known that alterations in the cell cycle control and the EMT constitute two important hallmarks of cancer cells [34]. ESRP1 and cyclin A2, respectively, have been identified as being vital for the regulation of EMT-associated splicing events or cell cycle progression related to cell proliferation [35,36]. The finding that ESRP1 directly regulates cyclin A2 expression at the post-transcriptional level will lead to a new understanding of the relationship between the EMT and the cell cycle, suggesting that they are not two separate physiological events, but may instead be related, with ESRP1 serving as a link between them in tumor cells. It is possible that upregulation of ESRP1 expression not only inhibits EMT, but also induces G1-phase arrest in cancer cells, and thus ESRP1 is likely to be an ideal candidate target for anti-cancer drugs, although more experiments will be needed to support this hypothesis. Furthermore, the molecular mechanisms by which TGF-β regulates the tumor cell cycle still remains unclear, and ESRP1 is an important target molecule downstream of the TGF-β signaling pathway [37]. As such, this finding will help to provide insight into the regulatory mechanisms of TGF-β-mediated cancer cells.

## 4. Materials and Methods

### 4.1. Cell Culture and Tissue Sample Collection

From July 2005 to July 2010, in the Department of Pathology of the Second People’s Hospital of Mudanjiang City, 44 samples of cervical squamous cell carcinoma were collected for analysis. Sample pathological diagnoses were established consistent with guidelines produced by the World Health Organization. HeLa and HEK293 cells from the Culture Collection of the Chinese Academy of Sciences (Shanghai, China) were cultured at 37 °C using DMEM (Gibco, NY, USA) supplemented with 10% FBS (Hyclone, Logan, UT, USA) and 1% penicillin/streptomycin (Gibco, NY, USA) in a humidified 5% CO_2_ incubator. We prepared stable HeLa-CYCA-Luc and HeLa-Luc cells as described previously [21].

### 4.2. Plasmids, Small Interfering RNA, and Transfection

To construct ESRP1 eukaryotic expression vectors (pcDEF-FLAG-ESRP1), the human ESRP1 cDNA was amplified via the following primers: 5′-GCGCGGATCCACCGCCATGGACTACAAGGACGATGATGACAAGGAATTCATGACGGCCTCTCCGGATTAC-3′ and 5′-GCGCTCTAGAGTTAAATACAAACCCATTCTTTGGGTAGAGTC-3′. Bam HⅠ and XbaⅠ were then used to cut the PCR product, which was then inserted into a pcDEF3 plasmid (Invitrogen, Carlsbad, CA, USA), with DNA sequencing used to confirm construct generation. The pEZX-MT06 plasmid was purchased from GeneCopoeia, Inc. (Guangzhou, China). GenePharma (Shanghai, China) synthesized siRNAs with the following sequences that were designed in a target-specific manner via BLAST searches: Human CDC20, 5′-AAUAUAUAUCCUCUGUTT-3′ (Sense), 5-CAGAGGAUAUAUAUUCCCTT-3′ (Anti-Sense); human cyclin A2, 5′-GGAUCUUCCUGUAAAUGAUTT-3′ (Sense), 5- AUCAUUUACAGGAAGAUCCTT-3′ (Anti-Sense); control scramble siRNA, 5′-UUCUCCGAACGUGUCACGUTT-3′ (Sense), 5′-ACGUGACACGUUCGGAGAATT-3′ (Anti-Sense). These siRNAs or plasmids were transfected as previously described [21].

### 4.3. RNA Isolation, Conventional RT-PCR, and Quantitative Real-Time RT-PCR (qRT-PCR)

An RNeasy mini kit (Qiagen, Valencia, CA, USA) was used to extract total cellular RNA based on provide protocols. The All-in-One First-Strand cDNA Synthesis Kit (GeneCopoeia, Guangzhou, China) was then used for cDNA reverse transcription based on provided protocols. Study primers were: Human ESRP1 (HQP013766, GeneCopoeia); human cyclin A2 (HQP021701, GeneCopoeia, Inc.); human GAPDH (HQP064347, GeneCopoeia); human TK-1, 5′-TTAACCTGCCCACTGTGCTGCCT-3′ (sense) and 5′-TTCTTGAAGTAGCAGAGCCGACAC-3′ (antisense); human GAPDH, 5′-TGT CGT GGA GTC TAC TGG TG-3′ (sense) and 5′-GCA TTG CTG ACA ATC TTG AG-3′ (antisense); human CD44, 5′-GCACTTCAGGAGGTTACATC-3′ (sense) and 5′-ACTGCAATGCAAACTGCAAG-3′ (antisense). The All-in-One Qpcr Mix Kit (GeneCopoeia) was used for all qRT-PCR reactions, following provided directions and utilizing an ABI 7500 FAST platform (Applied Biosystems, Foster, CA, USA). Conventional PCR was performed with 2.5 U LA Taq polymerase (Takara, Dalian, China). Thermocycler settings were: 95 °C for 3 min, 35 cycles of 95 °C 30 s, 55 °C 20 s, 72 °C 1 min, and then 72 °C for 5 min.

### 4.4. Assessment of Cell Proliferation 

Proliferation was gauged via in vitro bioluminescent imaging. We seeded HeLa-Luc cells in 96-well plates, and then imaged these cells at 48 h following ESRP1 or control vector transfection. Prior to imaging, wells were supplemented with 150 mg/mL (final concentration) D-Luciferin, and after 5 min a NightOWL LB 983 system (Berthold Technologies) was used for photon counting. Data were analyzed using IndiGO2 software.

### 4.5. Immunoblotting, Luciferase Assay, and Cell Cycle Analysis 

RIPA lysis buffer was used to extract protein from samples, which was then quantitated via BCA assay. Equivalent protein amounts were then separated using 10% SDS-PAGE gels, followed by transfer onto PVDF membranes. Membranes were immunoblotted with the following antibodies: Anti-β-Actin (Abcam, Cambridge, UK); anti-cyclin A2 (Abcam, Cambridge, UK); anti-CDC20 (Abcam, USA); and anti-FLAG (Abcam, Cambridge, UK). Protein bands were visualized using corresponding secondary antibodies, and then chemiluminescence was utilized to detect protein bands. Luciferase activity assay and cell cycle analysis were conducted as in previous studies [21].

### 4.6. Electrophoretic Mobility Shift Assay (EMSA)

After quantifying protein levels with a BCA kit (Pierce, Rockford, IL, USA), binding reactions were conducted in 15 μL binding buffer with labeled or unlabeled probes and purified recombinant 1500 ng FLAG-ESRP1 (Sino Biological Inc., Beijing, China). EMSA was conducted using the EMSA kit (Pierce, Rockford, IL, USA) based on provided directions. To check the specificity of the RNA-protein interaction, competition was performed using 50- and 150-fold quantities of cold oligonucleotides. Samples were then separated on 6% non-denaturing gels via electrophoresis, and the resultant complexes underwent transfer to a nylon membrane with a positive charge. Probe sequences were: Biotin RNA probes, biotin-GUUUUGCACUGGUGGUCUGUGUUCUG-biotin; cold RNA oligos, cold-GUUUUGCACUGGUGGUCUGUGUUCUG-cold.

### 4.7. RNA Immunoprecipitation Assay

RNA immunoprecipitation (RIP) assay was conducted with a Magna RIP™ RNA-Binding Protein Immunoprecipitation Kit (Merck Millipore, Bedford, MA, USA) based on provided directions. The anti-FLAG antibody and IgG (as a control) were used, and co-precipitated RNAs were detected by qRT-PCR or conventional RT-PCR. Primers were as follows: 5′-TGGTCTGTGTTCTGTGAATA-3′ (sense); 5′-TCTTGGATGCCAGTCTTAC-3′ (antisense).

### 4.8. Immunohistochemistry 

Immunohistochemistry was conducted as in previous studies [38]. Anti-human ESRP1 (Sigma, St. Louis, MO, USA) or Ki-67 (1:150) (Abcam, Cambridge, UK) were used for staining, after which two blinded pathologists independently scored all samples. Samples were scored based on nuclear ESRP1 or Ki-67 levels, with scores being determined based on a sum of nuclear staining intensities and positive staining area as a percentage, with the latter being determined in a manner consistent with previous studies [29].

### 4.9. Statistical Analysis

SPSS 15.0 and Graph Pad Prism 5.0 software were used for statistical analysis. Data are means ± standard error (SEM). Student’s *t*-test was utilized for all analyses, with *p* < 0.05 being the significance threshold. All experiments were performed at least three times to insure reproducibility of the results.

## 5. Conclusions

This study provides the first evidence that ESRP1 overexpression induces G1-phase cell cycle arrest via reducing the stability of the cyclin A2 mRNA, and inhibits cervical carcinoma cell proliferation. The findings suggest that the ESRP1/cyclin A2 regulatory axis may be essential as a regulator of cell proliferation, and may thus represent an attractive target for cervical cancer prevention and treatment.
